# Genome Studies on Nematophagous and Entomogenous Fungi in China

**DOI:** 10.3390/jof2010009

**Published:** 2016-02-05

**Authors:** Weiwei Zhang, Xiaoli Cheng, Xingzhong Liu, Meichun Xiang

**Affiliations:** State Key Laboratory of Mycology, Institute of Microbiology, Chinese Academy of Sciences, No. 3 Park 1, Beichen West Rd., Chaoyang District, Beijing 100101, China; davidbeckham19908@163.com (W.Z.); xiaoli0536@163.com (X.C.); liuxz@im.ac.cn (X.L.)

**Keywords:** fungal genome, biological control, nematophagous, entomogenous

## Abstract

The nematophagous and entomogenous fungi are natural enemies of nematodes and insects and have been utilized by humans to control agricultural and forestry pests. Some of these fungi have been or are being developed as biological control agents in China and worldwide. Several important nematophagous and entomogenous fungi, including nematode-trapping fungi (*Arthrobotrys oligospora* and *Drechslerella stenobrocha*), nematode endoparasite (*Hirsutella minnesotensis*), insect pathogens (*Beauveria bassiana* and *Metarhizium* spp.) and Chinese medicinal fungi (*Ophiocordyceps sinensis* and *Cordyceps militaris*), have been genome sequenced and extensively analyzed in China. The biology, evolution, and pharmaceutical application of these fungi and their interacting with host nematodes and insects revealed by genomes, comparing genomes coupled with transcriptomes are summarized and reviewed in this paper.

## 1. Introduction

Nematophagous fungi infect their hosts using traps and other devices such as adhesive conidia and parasitic hyphae tips [1,2]. Entomogenous fungi are associated with insects, mainly as pathogens or parasites [3]. Both nematophagous and entomogenous fungi are important biocontrol resources [1,3]. *Cordyceps* spp. are ancient resources in Chinese medicine [4]. Biological control is an economically and ecologically friendly approach for controlling nematode and insect pests that have a negative economic impact in agriculture [5]. Nematophagous and entomopathogenic fungi have been used with various strategies for almost a century and, for instance, mass production of entomopathogenic fungi were utilized for insect-pest control in the field while nematophagous fungi were released to the soil for nematode control [6,7]. There is also a long history of utilizing these fungi for the control of agricultural and forestry pests in China [8].

Among these fungi, *Arthrobotrys oligospora* (adhesive network traps) and *Drechslerella stenobrocha* (constricting ring trap) are well-known nematode trapping fungi in the Orbiliales (Orbiliomycetes and Ascomycota) [9,10,11]; *Hirsutella minnesotensis*, as a nematode endoparasite, responds to the natural suppressiveness of soybean cyst nematode [12,13,14]; *Ophiocordyceps sinensis*, a famous fungus used as a traditional Chinese medicine, belongs to Ophiocordycipitaceae, Hypocreales (Sordariomycetes, Ascomycota) [15]; *Cordyceps militaris* as medicinal fungus and *Beauveria bassiana* as biocontrol agent of insect are representatives of Cordycipitaceae, Hypocreales (Sordariomycetes, Ascomycota) [16,17]; and *Metarhizium* spp. in Clavicipitaceae, Hypocreales (Sordariomycetes, Ascomycota) are important entomopathogens [18]. The biology, evolution, interaction with hosts, biocontrol application for pests, and medicinal use of these fungi have been extensively studied by Chinese mycologists [19,20].

Recently, development of next generation sequencing technology allows the easy obtention of high quality eukaryotic genome sequences [21,22]. A number of nematophagous and entomogenous fungi have been genome sequenced. Genomic surveys have provided new strategies to answer biological issues of those fungi and their sophisticated mechanisms for interacting with their invertebrate hosts. A number of nematophagous and entomogenous fungi extensively studied in China have been genome sequenced by Chinese mycologists [9,10,13,15,16,17,18]. The approaches obtained from analysis of genomes, and comparing genomes and transcriptomes of these fungi, provide comprehensive understanding of their biology and interaction with invertebrate hosts, and furthermore, enhance their application in pest control and medicine utilization.

## 2. Nematophagous Fungi

**Figure 1:**
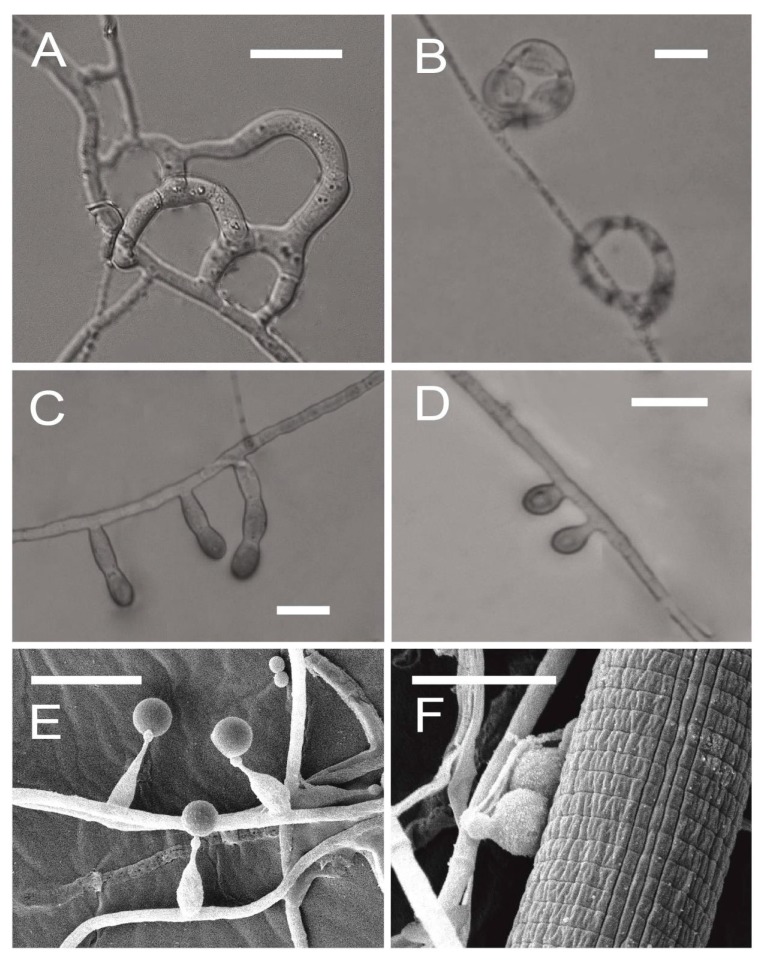
Morphology of traps and adhesive conidia produced by nematophagous fungi: (**A**) Adhesive nets (*Arthrobotrys oligospora*); (**B**) Constricting rings (*Drechslerella stenobrocha*); (**C**) Adhesive columns (*Gamsylella cionopaga*); (**D**) Adhesive knobs (*Gamsylella robusta*); and (**E**, **F**) Adhesive conidia (*Hirsutella minnesotensis*). Bars = 10 μm.

Nematophagous fungi can use specialized traps to capture nematodes, conidia to adhere to nematodes, hyphae tips to parasitize nematode females and eggs, or produce toxins to attack nematodes [2]. Nematode-trapping fungi and nematode endoparasitic fungi are two important groups of nematophagous fungi. Predation is one of the fungal life-strategies to destroy nematodes [1,23] by capturing nematodes with sophisticated trapping structures, including constricting rings and several other types of adhesive traps (sessile adhesive knobs, stalked adhesive knobs, adhesive nets, adhesive columns, and non-constricting rings) (Figure 1A–D) [24,25]. The traps are usually produced from hyphae and induced by nematodes, peptides, and nematode extracts [26,27]. Most of the nematode-trapping fungi belong to Orbiliales (Ascomycota). Among these fungi, *Arthrobotrys oligospora* forming adhesive nets was first genome sequenced to understand the molecular basis of the mechanisms for capturing nematodes (Table 1) [28,29]. A proposed model containing multiple fungal signal transduction pathways associated with diverse cellular processes such as energy metabolism, biosynthesis of the cell wall and adhesive proteins, cell division, glycerol accumulation, and peroxisome biogenesis [29]. The following sequenced genome by Chinese mycologists was *Drechslerella stenobrocha*, which forms constricting rings to actively capture nematodes. Comparative genomic analysis provided support for the hypothesis that nematode trapping fungi evolved from saprophytic fungi in a high carbon and low nitrogen environment and revealed the transition between saprophagy and predation for further understanding of the interaction between nematode-trapping fungi and nematodes [30].

**Table 1:** Available genomic sequences nematophagous fungi in China.

Features	Nematode-Trapping Fungi		Nematode Endoparasitic Fungi	Entomogenous Fungi				
	*Drechslerella stenobrocha*	*Arthrobotrys oligospora*	*Hirsutella minnesotensis*	*Metarhizium robertsii*	*Metarhizium acridum*	*Beauveria bassiana*	*Cordyceps militaris*	*Ophiocordyceps sinensis*
Assembled size (Mb)	29.02	40.02	51.4	39.04	38.05	33.7	32.2	~120
Protein-coding genes	7781	11,479	12,702	10,582	9849	10,366	9684	6792
Coverage (fold)	80×	-	128	100	107	76.6	147	241
Number of scaffolds	142	-	967	174	241	242	147	-
Scaffold N50 (kb)	434.4	-	382.4	1960	330	730	4550	-
G + C content (%)	52.5	45.2	52.1	51.49	49.91	51.5	51.4	46.1
Simple repeat rate (%)	0.92	-	1.33	0.98	1.52	2.03	3.04	37.98
TEs (%)	-	-	34.67	-	-	-	-	-
Gene density (genes per Mb)	268	271	247.1	271.1	258.8	308	257	87
Exons per gene	3.5	3.3	2.5	2.8	2.7	2.7	3.0	2.6
tRNA genes	82	149	145	141	122	113	136	-
References	Liu *et al.* 2014 [10]	Yang *et al.* 2011 [9]	Lai *et al.* 2014 [13]	Gao *et al.* 2011 [18]	Gao *et al.* 2011 [18]	Xiao *et al.* 2012 [17]	Zheng *et al.* 2011 [12]	Hu *et al.* 2013 [15]

Unlike nematode-trapping fungi, *Hirsutella* spp. are representatives of nematode endoparasitic fungi in Hypocreales (Ascomycota). *Hirsutella minnesotensis* and *Hirsutella rhossiliensis*, as parasites on cyst nematodes, can suppress the population of soybean cyst nematodes (SCN, *Heterodera glycines*) and are widely distributed in the northeast of China and Minnesota in the United States [12,29]. *H. minnesotensis* is the first genome sequenced nematode endoparasitic fungus [13]. It can parasitize nematodes by means of adhesive spores (Figure 1E,F). Genome of *H. minnesotensis* is different from those of nematode-trapping fungi but similar to those of entomopathogenic fungi. It has fewer genes encoding lectins for adhesion and glycoside hydrolases (GHs) for cellulose degradation, but more genes for protein degradation, signal transduction, and secondary metabolism [13].

### 2.1. Different Origin of Nematode-Trapping and Endoparasitic Fungi

As a special living strategy of fungi, the origin and development of predation are an essential and attractive topic in evolutionary biology of fungi. It was deduced that predation was evolved to obtain the nitrogen from nematode in the environments that are rich in carbon but poor in nitrogen [30]. Direct capture of nitrogen from small animals would provide predatory fungi with a competitive advantage over strictly saprophytic fungi [31]. A previous study based on the multigene phylogeny estimated that the lineage of nematode-trapping fungi in Ascomycota diverged at 246 million years ago (Mya), five million years after the Permian–Triassic extinction (251.4 Mya) [30,32]. Other clades of fungi that form adhesive traps diverged around 200 Mya, coinciding with the Triassic–Jurassic extinction (201.4 Mya) [30]. However, still little is known about the origin of predatory fungi for the lack of strong direct evidence.

**Figure 2:**
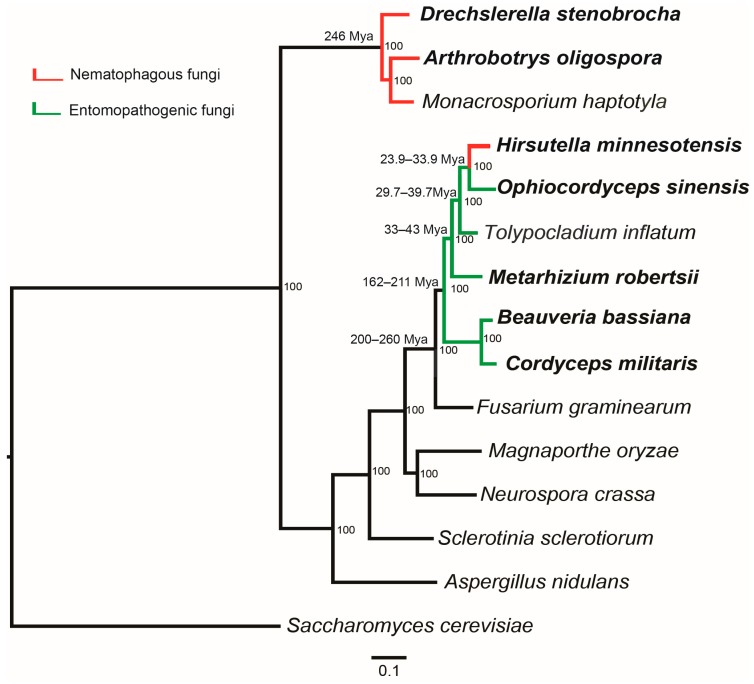
Genome-based phylogenetic tree of nematophagous fungi and other fungi. Bootstrap values and divergence time are indicated beside the nodes; color of branches corresponds to the lifestyle character states as follows: red, nematophagous fungi; green, entomopathogenic fungi; black, other fungi.

Currently, available genomic sequences of nematode-trapping fungi have made it possible for the evolutionary study at genomic level. Phylogenomic relationships constructed via the genomic sequences confirmed that both *D. stenobrocha* and *A. oligospora* are affiliated with the family Orbiliaceae (Figure 2) [9,10]. The analysis indicated that the nematode-trapping fungi were efficient saprobes. The identified large numbers of enzymes involved in the process of saprophytic degradation suggested that nematode-trapping fungi are more similar to fungal saprobes rather than animal pathogens or plant pathogens [10]. Among these enzymes, the largest number of proteins are carbohydrate binding module 1 (CBM1)-containing cellulases, previously found to be associated with cellulose degradation in the typical rot fungus (*Phanerochaete chrysosporium*) and the coprophilic *Podospora anserina* [33,34]. Thus, nematode-trapping fungi are suggested to be efficient saprobes and this provides more evidence for the hypothesis that nematode-trapping fungi might have originated in an environment rich in carbon resources but lacking nitrogen.

As a nematode endoparasite in the order Hypocreales, phylogenomic analysis indicated that *H. minnesotensis* is clustered to the caterpillar fungus *O. sinensis*, an insect pathogen. *H. minnesotensis* diverged from *O. sinensis* around 23.9–33.9 Mya and from *Tolypocladium inflatum* around 29.7–39.7 Mya (Figure 2) [13]. This suggests that the speciation of nematode endoparasites is independent with the mass extinction but more likely originated from entomopathogens.

### 2.2. Lectins and Other Adhesive Proteins Involved in Adhering to Nematode

One of the important mechanisms of fungi recognizing their nematode host is mediated by lectins. The traps of *A. oligospora* that are challenged by GalNAc (*N*-acetylgalactosamine) lost the ability to capture nematodes [35]. Other monosaccharaides such as glucose and mannose can also inhibit the capacity of the trapping-fungi to recognize nematodes [36]. However, the knocked out lectin-coding gene in *A. oligospora* did not affect the trapping ability or vegetative growth [37]. Moreover, lectins were not detected to be differentially expressed during nematode trapping [9]. Thus, the genomic and transcriptomic data did not support the previous hypothesis of lectin-mediated nematode recognition [9].

The lectin-coding genes identified in the genomes of nematode-trapping fungi suggest that lectins might play a more important role in the adhesive networks than in the constricting rings. More putative lectin-coding genes were identified in the genome of *A. oligospora* than in *D. stenobrocha*, especially in the families of fucose-specific and H-type lectins [9,10]. It is also possible that the recognition of nematodes is executed by multiple lectin-encoding genes, and this would explain why the deleted lectin gene did not affect the nematode capture capacity of the fungi. Compared with trapping fungi, H-type and fucose-specific lectins are absent in the *H. minnesotensis* genome and this would indicate that nematode endoparasitic fungus has evolved a different mechanism to adhere to the nematode hosts [13]. Furthermore, expression of lectin-encoding genes of *H. minnesotensis* is significantly up-regulated during the infection to nematode. Based on the genomic data, pretreating the nematodes with different lectins and their mixtures can also decrease the adhesion rate of *H. minnesotensis* [13]. Coupled with previous studies, it is still possible that lectin is important to mediate the nematode recognition by fungi.

In addition to the lectins, other adhesive proteins were also suggested to contribute to the adhesion of fungi to nematodes. For instance, numerous CFEM-containing proteins have been identified in the both nematode-trapping and endoparasitic fungal genomes [9,10,13]. The function of CFEM-containing adhesive proteins was confirmed by the transcriptomic analysis of *H. minnesotensis* and the qPCR analysis of *A. oligospora* during nematode infection [9,13]. Other supposedly adhesive proteins such as GLEYA-containing proteins have also been found in the nematode-trapping fungi [9,10]. However, most of the adhesive proteins are still functionally unknown and should be investigated in detail in the future.

### 2.3. Formation of Traps and Infection Pegs

Traps and infection pegs are the only specialized hyphae by which the nematode-trapping and endoparasitic fungi infect and penetrate nematode cuticle. The processes to form these sophisticated hyphal structures are very complicated and poorly understood. Comparing the expressing patterns between the saprophytic and predatory lifestyles based on the proteomic analysis of *A. oligospora* predicted from its genome indicated that multiple fungal pathways are proposed to be involved in the trap formation of *A. oligospora*, including mitogen activated protein kinase (MAPK) pathway, cell division, energy metabolism, biosynthesis of the cell wall and adhesive proteins, glycerol accumulation, and peroxisome biogenesis [9]. Similar results are found from the transcriptomic analysis of *D. stenobrocha* during the trap formation and infection of nematodes. In addition, Zn(2)-C6 type transcription factors might also be associated with signal transduction [10]. Though the formation of infection pegs of *H. minnesotensis* is quite different from the traps of nematode-trapping fungi, the protein kinase C (PKC) signal transduction pathway is shared by both predatory fungi and endoparasites. Meanwhile, cyclic AMP-protein kinase A pathway and STM1-like G protein-coupled receptor (GPCR) responsible for filamentation are also thought to be involved in nematode infection [13].

### 2.4. Virulence Factors Associated with Nematode Infection

#### 2.4.1. Extracellular Enzymes

Penetrating and digesting the nematode cuticles play key roles during the fungal infection and the consumption of nematodes. Enzymes such as proteases, collagenases, and chitinases are the virulence factors of nematophagous fungi [9]. Serine proteases are the extensively studied enzymes and, more than 20 of them have been cloned or purified from the nematophagous fungi and are considered as virulent factors [20,38,39]. In addition, the improved pathogenicity by overexpressing serine protease also confirms its role in virulence function [40].

Genome sequencing has revealed a large number of proteases associated with the degradation of the nematode cuticle. Subtilisins, as one of the most important families belonging to serine proteases, namely S08, are abundant in the nematophagous fungi and totally 43 in *A. oligospora*, 18 in *D. stenobrocha*, and 21 in *H. minnesotensis* have been annotated [9,10,13]. However, only two subtilisin-coding genes in *A. oligospora* and one in *D. stenobrocha* were found up-regulated when they were challenged by nematode or nematode extracts (NE) [9,10]. Although five subtilisins were up-regulated that might be associated with infection processes of *H. minnesotensis*, the function of other subtilisins predicted in the genomes of nematophagous fungi is still unknown [13]. Besides the subtilisins, other proteases are highly expressed during the nematode infection, such as protease S28 (DRE_00914) and S33 (DRE_00834) in *D. stenobrocha* as well as endopeptidases (M4 and M43) and exopeptidases (M14 and M28) in *H. minnesotensis* [10,13]. This indicates that the degradation of nematode cuticles is not only associated with subtilisins but also a series of other secreted enzymes. Further investigation on these secreted enzymes should be performed to fully understand the molecular mechanisms of fungi infecting nematodes and will provide new insight to improve the pathogenicity of nematophagous fungi.

#### 2.4.2. Secondary Metabolites

It has been long known that most of the compounds against nematodes are produced by toxin-producing fungi that can use them to immobilize nematodes prior to infection [41]. Although the secondary metabolism was investigated more than thirty year ago in the nematode-trapping fungi, only 10–19 genes involved in secondary metabolisms are found in trapping fungi [42,43]. Genomics provides a powerful tool for the study of secondary metabolites: consequently 94 secondary metabolite gene clusters and 101 core genes have been recently identified in the *H. minnesotensis* genome while only one to four core genes were found in the trapping fungi [13]. The transcriptomic profiling of *H. minnesotensis* has indicated that the genes encoding secondary metabolites are up-regulated before and during nematode penetration, and mycelial growth for consuming the nematode body, which suggested those compounds could be virulent factors [13]. Further investigation on the secondary metabolites of *H. minnesotensis* found that some compounds have nematicidal activity [44].

## 3. Entomopathogenic Fungi

Entomopathogenic fungi are natural enemies to manage insect pests in the ecosystems and more than 700 species have been discovered so far [45]. Some of these fungi have been developed as biological control agents and widely used to control insect pests [46]. *Metarhizium robertsii* and *B. bassiana* are two well-known biocontrol agents that have been approved by the US Environmental Protection Agency (EPA). Both species are the members of Hypocreales (Ascomycota), an order including a large number of pathogenic fungi of insects, plants, and other fungi, as well as medicinal fungi [47,48]. The biology of those two fungi and their interaction with host insects have been extensively studied and most of the studies focus on the pathogenic processes. Genes involved in regulating expression of virulent factors, the formation of infection structures, and adhesive proteins have been functionally identified in *Metarhizium* spp. [49,50,51]. Similarly, virulence-related genes have been also characterized in *B. bassiana*, such as MAP kinases and the neuronal calcium sensor contributing to virulence [52,53,54]. Likewise, the secondary metabolites with various biological activities such as insecticidal, antibiotic, and anti-tumor have also been investigated in these two fungi [55,56,57,58]. Therefore, *M. robertsii* (formerly known as *M. anisopliae* var. *anisopliae*), and *M. acridum* are distributed worldwide and extensively studied entomopathogenic fungi. *Cordyceps* spp. have been used as traditional Chinese medicine for centuries and the fungal products are a big industry in China. A number of medicinal and health products have been developed and extensively commercialized from natural *C. militaris*, *O. sinensis* (anamorph: *Hirsutella sinensis*), and other *Cordyceps* spp. [4]. The market demand of *O. sinensis* is huge while the resource in nature is limited and the artificial cultivation of the fruit body has not been reported. Some ghost moths have been successfully raised in large scale while the infection rate of the ghost moths by *H. sinensis* was quite low and this might due to the poor understanding of its infective mechanisms [4]. These fungal species have been genome sequenced by Chinese mycologists [15,16,17,18]. Several biological issues have been comprehensively understood from the genome data.

### 3.1. Divergence and Origin of Insect Pathogens

Hypocreales is one of the most important orders in Ascomycota and its members include grass endophytes as well as parasites of plants, insects, and fungi [59]. The entomopathogenic fungi with genome sequenced mainly belong to this order (Figure 2) including *C. militaris* and *B. bassiana* in Cordycipitaceae, *Metarhizium* spp. in Clavicepitaceae, and *O. sinensis* in Ophiocordycipitaceae [47]. Hypocrealean species exhibit a broad range of nutritional modes based on plants, animals, and other fungi, while the Hypocreales order is suggested to origin from the plant-based nutritional mode [59]. *C. militaris* diverged 166–211 Mya, closely related to the wheat pathogen *Fusarium graminearum* (diverged 200–260 Mya) [60]. Compared with *C. militaris*, the divergence of *Metarhizium* spp. (33–43 Mya) is much later while *O. sinensis* (diverged 23.9–33.9 Mya) is closely related to nematophagous *H. minnesotensis* [13,15,18]. Thus, *O. sinensis* was hypothesized to origin from *C. militaris* and *H. minnesotensis* might evolve from entomopathogens [15].

*Metarhizium* spp. are closely related with grass endophytes *Epichlöe* spp. [61] and are most abundant in grass root soils [62,63], indicating that the habitat of some *Metarhizium* spp. is not only insects but also the rhizosphere. The adhesive proteins, MAD1 and MAD2, as well as other genes involved in colonizing the root rhizosphere are presented in *Metarhizium* spp. [50,64]. Almost all families of the enzymes involved in plant cell wall degradation are presented in the genomes of *Metarhizium*, even some of them are absent in the plant colonizer *Trichoderma reesei* [18,65]. By screening the genes against the pathogen–host interaction base (PHI) among *Metarhizium* and plant-associated *F. graminearum* and *Magnaporthe oryzae*, PHI genes related with plant host are identified to be highly homologous shared by these fungi living on different strategies, whereas fewer *Metarhizium* orthologs were shared with animal pathogen *Candida albicans* [18]. Although *Metarhizium* spp. are well recognized as insect pathogens, they are more closely related to plant pathogens than to animal pathogens, suggesting their origin from plant-associated fungi. *Metarhizium* spp. have been found to be more abundant in agricultural than in forestry fields and the application of *M. robertsii* has shown beneficial effects on plants [66,67].

### 3.2. Host Recognition and Signal Transduction

Recognition and adaption to nutrient availability of hosts are mediated by signal transduction. Pth11-like GPCR (PHI-base acc: 404) was identified in *Magnaporthe* to mediate cell responses to inductive cues [68]. By comparing the transcriptional responses to hosts, it was found as a key signal receptor widely shared by the entomopahogens and up-regulated in *M. robertsii*, *M. acridum*, and *B. bassiana* during the host recognition stage [17,18]. However, it was not detected to be differentially expressed during the infection to nematode in *H. minnesotensis* [13]. The PKC belonging to the MAPK pathway signal transduction pathway is strongly activated during the infection of both nematodes and insects [9,10,13,17,18]. Moreover, STM1-like GPCR was characterized to adapt to nitrogen starvation in fission yeast *Schizosaccharomyces pombe* [69]. The STM1-like GPCRs were up-regulated during the whole pathogenic processes of *H. minnesotensis* as well as during the infection of *Metarhizium* spp. [9,10]. Thus, the signal transductions during the infection of nematode and insect are suggested to be analogous whereas differ in the recognition processes.

### 3.3. Secreted Enzymes Involved in Penetration of Insect Cuticles

To penetrate the protein-chitin rich insect cuticles and utilize host tissues as nutritional resources, entomopathogenic fungi were characterized to secrete large numbers of degradation enzymes as virulence factors. Representatively, *M. robertsii* and *M. acridum* have more genes encoding secreted proteases than any other sequenced fungus (93) with the number of 132 and 104, respectively [18]. The number of glycoside hydrolases (GH) possessed by *M. robertsii* (156) and *M. acridum* (140) is close to the average for plant pathogenic fungi (150) [18]. Similarly, totally 145 GHs are produced by *B. bassiana* [17]. However, *O. sinensis* has only 66 GHs and misses the enzymes devoted to degradation of plant materials [15].

Though the entomopathogenic fungi of Cordycipitaceae and Clavicipitaceae diverge into quite different lineages, similar enzymes are shared in these fungi suggesting that they all have potential targets in insect hosts [16,17]. Consistent with the previously characterized enzymes, numerous subtilisins, aspartyl proteases, chitinases, and lipases have also been identified as key enzymes responsible for the degradation of protein-chitin rich insect exoskeleton [18]. Chitin is a major component of insect cuticle that is more crucial for entomopathogens to penetrate [70]. Among the putative GHs, GH18 chitinases are predicted to be involved in the digestion of insect cuticle chitin [71]. The necessity to degrade chitin is reflected in the larger number of chitinases represented in *Metarhizium* (30 in *M. robertsii* and 21 in *M. acridum*), *B. bassiana* (20), and *C. militaris* (20) than in the compared plant pathogens (average 11) [16,17,18]. Phylogenetic analyses of the chitinase coding genes revealed that the duplication events occurred after the speciation of *B. bassiana* and *C. militaris*, *Metarhizium* spp., and, *Trichoderma* spp., respectively [17]. Thus the abundance of chitinases might be due to convergent evolution [17]. In contrast, only nine chitinase coding genes were identified in *O. sinensis*, indicating the weaker capacity to breach insect cuticle [15].

Proteases are another kind of enzymes predicted to degrade the protein-chitin rich insect cuticle. The entomopathogenic fungi code for large numbers of proteases required during infection. The most significant expansion of proteolytic enzymes occurs in the trypsin family (protease S01) [17,18]. Six to ten times the trypsins were predicted in the genomes of entomopathogens (12–32) than that in plant pathogens (4 or less) [16,17,18]. The distribution of trypsins might be caused by the specific duplication detected in the *M. robertsii* genome and horizontal transfer in the *B. bassiana* genome from bacteria [17,18]. In addition, the vital function was also indicated by the high expression during the infection process of *M. robertsii* [18]. Consistent with the overall loss of enzymes in the *O. sinensis* genome, only two trypsins were identified [15].

Subtilisins belonging to protease S08 are shared by entomopathogens and nematophagous fungi as virulence factors. A total of 55 subtilisins in *M. robertsii* and 43 in *M. acridum* were identified, similar to that in *B. bassiana* (42), while there were more than that in *O. sinensis* (17) [15,17,18]. However, similar to nematophagous fungi, most of the subtilisins were not detected to be abundantly expressed [10,13]. Overall, the reduction of virulence factors in *O. sinensis* suggests that *O. sinensis* might be selected to avoid host defenses, while other entomopathogens evolved to be highly parasitic to hosts [15].

### 3.4. Secondary Metabolites

Secondary metabolites in entomogenous fungi have been extensively investigated. They are not only insecticidal such as destruxins but also active against bacteria or tumor cells [55,58]. Cordycepin is one of the main active components in *C. militaris* for medicinal use [72]. Other compounds, such as bauvericin, tenellin, bassiatin, oosporein, and destruxin have already been identified, however, the gene clusters that involved in their biosynthesis were rarely known before the genomic sequencing [73,74,75,76]. On the other hand, secondary metabolites are also involved in the host range of entomopathogenic fungi. *M. roberstii* as a broad host range fungus has a larger potential to produce secondary metabolites for killing the hosts via toxins and grow saprophytically comparing to the acridid-specific *M. acridum* to kill the hosts by systemic infection [77]. More putative core genes associated with the production of secondary metabolites have been identified in *M. roberstii* (43 core genes) than in *M. acridum* (20 core genes). [18]. Gain and loss of destruxin gene clusters is closely related to the host specificity in *Metarhizium* [78]. Some of the genes involved in insecticidal activities are highly homologous, and *M. robertsii* also possesses a putative bassianolide synthetase, a crucial virulence factor identified in *B. bassiana* [18]. In agreement with its use as a medicinal fungus, genes encoding mycotoxins are not present in *C. militaris*, whereas they are abundant (total number of 45 core genes) in *B. bassiana* [16,17]. The adaptation of pathogenesis and human utilization of entomogenous fungi then could be associated with their secondary metabolites.

### 3.5. Mechanisms of Fungal Pathogen Speciation and Host Adaptation

Since genomic data have provided the comprehensive understanding on mechanisms of fungal pathogenicity against insect hosts, comparing the fungi with narrow and wide host ranges provided new insights into the mechanisms of host adaption. Typically, gene expansion is significant in the wide host range fungi. For instance, the wide host range fungi *M. robertsii* and *B. bassiana* have more proteases with a higher proportion in the families of trypsins (average 28) and subtilisin (average 49) than in the narrow host range fungi *M. acridum* and *C. militaris* (averaged 14 trypsins and 43 subtilisins) [16,17,18]. Gene duplication or horizontal transfer might lead to the expansion of proteases as virulence factors [18]. At the same time, the lineage-specific expanded proteases might be responsible for the adaptation to a broad host range [17,18].

The 12 *Metarhizium* species described so far include those with wide insect host range, transitional species, and those with specialized hosts, providing excellent materials to research the speciation and evolution of fungi interacting with host insects [79,80,81,82]. Seven *Metarhizium* species were genome sequenced with different host ranges. Significantly, more proteins involved in both primary and secondary metabolism were identified in the species with wide insect host range than the specialist species (*M. album* and *M. acridum*) and these proteins could contribute to virulence and host range through production of mycotoxins or detoxification of host metabolites [79]. On the other side, the genes as virulence factors in *Metarhizium* spp. including serine proteases, chitinases, and CFEM domain-containing proteins as virulence determinants were detected to be positively selected, suggesting the *Metarhizium* spp. are undergoing rapid evolution during the host adaption [79].

*O. sinensis* can be found as a caterpillar fungus in Tibetan Plateau alpine ecosystems and is highly specialized to infect moth larvae (caterpillar fungus). The dependence on the nutritional insect-based resources leads to an overall loss of carbohydrate degrading enzymes with fewer glycoside hydrolases [15]. The genome is shaped by retrotransposon-driven expansions which is corresponding to the close interaction with host. The changes in gene content are very different from those species of *Cordyceps* spp. and *Metarhizium* spp. [15,16,18]. The entomopathogens provide a trade-off between the advantages of increased genetic variation and deletion of genes dispensable due to a specialized pathogenic lifestyle [15].

## 4. Conclusions

The improvements of next generation sequencing technologies of the high quality eukaryotic genome sequences have led to the availability of a large number of fungal genomes. Based on fungal genomics, the biology of nematophagous and entomogenous fungi is much more comprehensively understood, including the origin and evolution, mechanisms of interaction of the fungus and invertebrate, host specificity, and secondary metabolites. Transcriptomics and proteomics are essential supplements for genomics. “-Omics” have been a powerful tool for biological and ecological research. The following main points should be emphasized based on “-omics” in the future studies for nematophagous and entomogenous fungi.

1. Nematophagous and entomogenous fungi include many important species that impact ecology and human life. Origination and differentiation of some species such as *O. sinensis* or *H. minnesotensis* have been studied based on multigene analysis [83,84]. Availability of *de novo* genome sequences of these important species has made it possible for population genomics analysis that could provide a more comprehensive understanding of their origination, differentiation, and speciation.
2. Phylogeny based on single gene or multigenes has been extensively applied in the systematics and evolution of fungi. Origination and evolution of trapping devices of nematode-trapping fungi have been deduced to result from the mass extinct events based on the multigene analysis [25,30]. Since a number of fungal genomes have been sequenced, phylogenomics has become a new strategy for the systematic and evolutionary study.
3. Many nematophagous and entomopathogenic fungi have been developed/are being developed as biocontrol agents. The molecular mechanisms of fungi interacting with nematodes and insects are essential for utilization of these fungi in agricultural pest management. “-Omics” should be more efficient for finding and identifying the functional genes and pathways involved in the interaction between fungi and host pests that will help in the development of biocontrol agents.
4. Secondary metabolites are not only involved in the interaction between fungi and their host pests, but also the precursors to develop nematicides, insecticides and drugs. Destruxins were identified in 1990s while their biosynthetic puzzle was not solved until the genomes of *Metarhizium* spp. were sequenced [75,76]. “-Omics” have provided an efficient strategy to decipher and identify gene clusters encoding secondary metabolites.
